# Hepatic encephalopathy in cirrhosis and alcohol dependence: complex clinical challenges and multidisciplinary management

**DOI:** 10.1192/j.eurpsy.2024.1016

**Published:** 2024-08-27

**Authors:** C. Alario-Ruiz, B. Arribas-Simon, P. Martinez.Gimeno, M. Calvo-Valcarcel, O. Martin-Santiago

**Affiliations:** Hospital Clinico Universitario, Valladolid, Spain

## Abstract

**Introduction:**

Liver cirrhosis, a chronic liver disease, can be closely linked to chronic alcohol abuse, posing a significant medical challenge. Hepatic encephalopathy (HE), a neuropsychiatric condition resulting from liver dysfunction, commonly occurs in cirrhotic patients due to the accumulation of neurotoxic substances like ammonia and manganese in the body. Managing cirrhosis and alcohol addiction is crucial to enhancing the quality of life for these patients, as HE can manifest in various ways and with varying degrees of severity.

**Objectives:**

To emphasize the importance of recognizing and treating hepatic encephalopathy as a potential complication of liver cirrhosis and sedatives during alcohol withdrawal.

**Methods:**

We compiled clinical data, medical history, neuroimaging tests, and therapeutic interventions applied.

**Results:**

A 55-year-old man with a complex medical history, including Child-Pugh B liver cirrhosis, portal hypertension, hypertension, diabetes mellitus, and chronic alcohol abuse with numerous prior hospitalizations for acute pancreatitis and severe head trauma related to alcohol consumption, presented to the emergency department with symptoms of alcohol withdrawal and suicidal thoughts, leading to lorazepam administration and a recommendation for admission to a specialized Therapeutic Community. After 72 hours, he developed hepatic encephalopathy with symptoms such as confusion, sleep disturbance, sweet-smelling breath, abnormal hand movements, conjunctival icterus, and urinary difficulties.

An EEG revealed a globally attenuated and disorganized bioelectrical activity with triphasic waves. The magnetic resonance imaging showed signs of hepato-cerebral degeneration, including T1-weighted hyperintensity in the lentiform and mesencephalic nuclei due to manganese deposition. Treatment was adjusted to reduce sedative use, and therapy with Rifaximin and Lactulose was initiated to control blood ammonia levels. After a week, the patient exhibited significant neurological improvement, underscoring the importance of appropriate management in patients with hepatic encephalopathy related to liver cirrhosis and chronic alcohol abuse.

**Conclusions:**

This case underscores the complexity of HE in patients with liver cirrhosis and alcohol dependence. HE can present in various ways, from subtle symptoms to severe episodes of confusion and coma. Findings on EEG, such as triphasic waves, are characteristic of HE and reflect brain dysfunction. Furthermore, manganese accumulation in the brain, as evidenced by magnetic resonance imaging, may contribute to neurological symptoms in cirrhotic patients. In this context, the early recognition and multidisciplinary treatment are emphasized to improve the quality of life and prevent the progression of this neuropsychiatric complication. EEG and magnetic resonance imaging findings play an essential role in the evaluation of these patients.

**Disclosure of Interest:**

None Declared

